# Metacognitive Therapy for Social Anxiety Disorder: An A–B Replication Series Across Social Anxiety Subtypes

**DOI:** 10.3389/fpsyg.2018.00540

**Published:** 2018-04-12

**Authors:** Henrik Nordahl, Adrian Wells

**Affiliations:** ^1^Department of Psychology, Norwegian University of Science and Technology, Trondheim, Norway; ^2^St. Olavs Hospital, Nidaros DPS, Trondheim University Hospital, Trondheim, Norway; ^3^Division of Psychology and Mental Health, School of Health Sciences, Faculty of Biology, Medicine and Health, The University of Manchester, Manchester Academic Health Science Centre, Manchester, United Kingdom; ^4^Greater Manchester Mental Health NHS Foundation Trust, Prestwich, United Kingdom

**Keywords:** metacognitive therapy, social anxiety disorder, social phobia, case-series, metacognition

## Abstract

Cognitive behavioural therapy (CBT) is the treatment of choice for Social anxiety disorder (SAD). However, factors additional to those emphasised in CBT are the primary cause of psychological disorder according to the metacognitive model. Metacognitive Therapy (MCT) aims to target a perseverative thinking style named the cognitive attentional syndrome and its underlying metacognitive beliefs (beliefs about cognition). The present study aimed to explore the effects of generic MCT for SAD. Treatment related effects were evaluated using direct replication single case (A–B) methodology across three patients with different subtypes of SAD; performance type, generalised and generalised plus avoidant personality disorder, representing increasing SAD severity/complexity. All patients responded during treatment and achieved substantial symptom reductions which were largely maintained at 6 months’ follow-up. Metacognitive therapy appears to be a suitable treatment and was associated with positive outcomes for patients with different presentations of SAD.

## Introduction

Social anxiety disorder (SAD) or Social phobia is characterised by a marked or intense fear of social situations in which the individual may be scrutinised by others (American Psychiatric Association, 2013). SAD can be viewed on a severity continuum ranging from the performance type characterised by fear of negative evaluation in specific performance situations, to the generalised type characterised by fear of negative evaluation in most social situations, to the generalised type with comorbid Avoidant personality disorder (AvPD) (Bögels et al., 2010; American Psychiatric Association, 2013; Heimberg et al., 2014).

The treatment of choice for SAD is Cognitive therapy (CBT) based on the model by Clark and Wells (1995; National Institute for Health and Care Excellence, 2013) and it has been found to be superior to other psychological treatments and drugs (Mayo-Wilson et al., 2014). The model Clark and Wells (1995) draws on concepts from cognitive (e.g., Beck, 1976) and metacognitive (Wells and Matthews, 1994) theory. It proposes that on entering social situations people with social anxiety experience negative automatic thoughts and shift attention to self-focus on a biassed and distorted inner image of the self. Safety behaviours are used to deal with negative beliefs about how one appears to others but impair performance and increase self-focused attention. In addition, anticipatory worry and post-event rumination before and after social encounters contributes to problem maintenance. This pattern of processing can be traced back to underlying negative beliefs and assumptions about the social self (e.g., “I’m boring”).

A conceptual feature with the model Clark and Wells (1995) is that whilst it draws on different theoretical frameworks, it places cognition rather than metacognition in centre stage. For example, it argues that schemas or negative beliefs (e.g., “I’m a failure”) give rise to self-processing and social anxiety. However, the metacognitive model argues that metacognitive beliefs, beliefs about cognition (e.g., “I cannot control my thinking”), contribute most to disorders including social anxiety (Wells and Matthews, 1994). Furthermore, in the cognitive model the emphasis is on challenging the validity of negative social cognitions whilst in MCT the focus is on controlling cognition and modifying metacognitive beliefs.

In accordance with Wells’ (2000) metacognitive therapy approach, two studies (Wells and Papageorgiou, 2001; Nordahl et al., 2016b) have shown that a briefer and more metacognitive focused intervention can be highly effective and time efficient. However, these studies left out several important components which are emphasised in the metacognitive model (Wells and Matthews, 1994, 1996) and retained some of the cognitive components of the Clark and Wells (1995) treatment. For example, case-formulations were based on the CBT model, there was some work on testing negative thoughts (even though social beliefs were not challenged). However, more recent research on the relative contribution of social phobic beliefs (cognitive beliefs) and metacognitive beliefs in a social anxiety context has shown that metacognitive beliefs but not social phobic beliefs predict symptom improvement following treatment of social anxiety disorder (Nordahl et al., 2017), work status in high socially anxious individuals (Nordahl and Wells, 2017a), and depression symptoms in patients with social anxiety disorder (Nordahl et al., 2018). Therefore, testing of whether a purer metacognitive treatment can be applied and whether positive effects are associated with it is a greater priority.

According to the metacognitive model (Wells and Matthews, 1994), all psychological disorders are intensified and maintained by a thinking style called the *cognitive attentional syndrome* (CAS; Wells, 2009) consisting of worry/rumination, threat monitoring and maladaptive coping behaviours. Maladaptive metacognitive beliefs, i.e., beliefs about cognition, give rise to the CAS which in social anxiety take the form of negative metacognitive beliefs (“Worry is uncontrollable”), positive metacognitive beliefs (“focusing on an inner image of myself helps me avoid making a bad impression”) and judgements of cognitive confidence (“When I am under pressure, I lose my grip on thinking”) (e.g., Nordahl et al., 2016a, 2017; Gkika et al., 2017; Nordahl and Wells, 2017b). Metacognitive therapy (MCT; Wells, 2009) was developed to reduce the CAS and to modify underlying maladaptive metacognitive beliefs. MCT has been found to be an effective treatment for depression and several anxiety disorders (Normann et al., 2014), but has yet to be evaluated in its purer form in SAD.

We therefore aimed to conduct a preliminary investigation of the efficacy of MCT for SAD using single case methodology. Following the generic MCT conceptualization and treatment structure (Wells, 2009), we aimed to test if MCT could be applied using a single-case replication methodology that spanned cases of increasing complexity. Such an approach constitutes a systematic replication (Barlow and Hersen, 1984) and the search for cases in which the treatment may not work.

## Materials and Methods

### Design

In order to examine the effects associated with MCT for SAD, a single case series using an A–B design with follow up was implemented. This study was carried out in accordance with the recommendations of the Regional committees for medical and health research ethics in Norway with written informed consent from all subjects. All subjects gave written informed consent in accordance with the Declaration of Helsinki. The protocol was approved by the Regional committees for medical and health research ethics (reference number: 2015;1794). Replication across three patients with different SAD presentations begins to establish the generalizability of treatment effects across the disorder. This is particularly important in SAD as the disorder is found on a severity continuum. All patients were assigned to no-treatment baselines of a minimum of 3 weeks (with the option of extension if required) to establish stability in the primary outcome measure; the fear of negative evaluation (FNE: Watson and Friend, 1969). No therapeutic input occurred during the baseline period, but there was contact over telephone to ensure that the patients completed the self-report measures. Following the baseline period, eight sessions of MCT were delivered weekly with each treatment session lasting between 45 min and 1 h. Patents were followed up 6 months after treatment, and no additional treatment was delivered during the follow-up period.

### Participants

The first three patients with different presentations of social anxiety consecutively referred to the university outpatient clinical, Department of psychology, Norwegian University of Science and Technology, were included in the case series. Patients were assessed using the Structured Clinical Interview for DSM-IV Axis I Disorders (SCID-I/P; First et al., 1997b) and Axis II personality disorders (First et al., 1997a). The inclusion criteria were; (1) a primary diagnosis of SAD, (2) 18 years old or above, and (3) signed written informed consent in accordance with the Declaration of Helsinki. The exclusion criteria were; (1) concurrent psychological or drug treatment, (2) evidence of psychotic or organic illness, (3) the presence of cluster A or B personality disorder, (4) actively suicidal, or (5) substance or alcohol dependence.

#### Patient 1

Patient 1 was a 24-year old single woman struggling with the performance type of SAD for the past 4 years. In particular, making presentations as part of her studies was the major problem. She believed that she looked like “a patient with an epileptic seizure” while holding presentations because of conspicuous shaking. Furthermore, the patient described that she was anxious and worried a couple of weeks before, and for days after, presentations, leading to poor quality of life. Patient 1 had no comorbid diagnosis, and had never before had psychological treatment.

#### Patient 2

Patient 2 was a 70-year old retired man who presented with generalised SAD. He had been struggling with social anxiety since adolescence, and described conspicuous “shaking” as his primary symptom in social situations. He had psychological treatment for social anxiety 25 years before referral to the clinic, and experienced a brief non-lasting symptom improvement from that. The patient had for many years endured most social situations with support from his wife. However, some months ago he started to have panic attacks before social situations and therefore started to avoid most of them. The patient felt that his social anxiety now stopped him from having a normal life together with his wife, and that he wasn’t able to break out of this vicious cycle.

#### Patient 3

Patient 3 was a 27-year old single woman who presented with generalised SAD, Avoidant personality disorder, and a recurrent depressive disorder, currently moderately depressed. She had been suffering with social anxiety since she started primary school, and had dropped out from her studies several times because of social anxiety. In addition to being afraid of social embarrassment, the patient presented with low self-esteem and a profound tendency to avoid. The patient reported several depressive episodes, the current lasting for 6 months. She had previously had unspecific psychological treatment which had ended 2 years before referral to the clinic. She reported that she had not found her previous treatment helpful.

### Measures

The Fear of Negative Evaluation scale (FNE; Watson and Friend, 1969) is a 30-item measure of apprehension and anxiety over anticipated social evaluations. This measure uses a true-false scale and has shown good internal consistency (α = 0.94) and test–retest reliability (*r* = 0.78) (Watson and Friend, 1969). FNE has a range from 0 to 30, high scores indicating higher levels of social anxiety.

The Social Avoidance and Distress scale (SAD; Watson and Friend, 1969) is a 28-item measure of distress in social situations and avoidance, using a true–false scale. Its internal consistency has been found excellent (α = 0.94) and its test–retest reliability ranged from 0.68 to 0.79 (Watson and Friend, 1969). SAD has a range from 0 to 28, high scores indicating higher levels of social anxiety.

The Social Interaction Anxiety Scale (SIAS; Mattick and Clarke, 1998) is at 20-item scale that measure fear of and responses to social interactions. It has shown high internal consistency (α = 0.93) and test–retest reliability (0.92). SIAS has a range from 0 to 80, high scores indicating higher levels of social anxiety.

Beck Anxiety Inventory (BAI: Beck et al., 1988a) is a 21-item self-report scale designed to assess the severity of somatic and cognitive anxiety symptoms over the previous week. Scores range from 0 to 63, high scores indicating higher levels of social anxiety. BAI has high internal consistency (α = 0.92) and good test–retest reliability (0.75) (Beck et al., 1988a).

Beck Depression Inventory (BDI: Beck et al., 1961) is a 21-item self-report scale assessing current level of depression. BDI has a range from 0 to 63, high scores indicating higher levels of depression. The BDI has high internal consistency (α = 0.86) and the test–retest reliability has been reported as more than 0.60 (Beck et al., 1988b).

The MCQ-30 (Wells and Cartwright-Hatton, 2004) is a 30-item self-report scale measuring beliefs about thinking. Responses are required on a four-point scale, and the scales total score range from 30 to 120. The measures consist of five subscales measuring positive beliefs about worry; negative beliefs about the uncontrollability of thoughts and corresponding danger; cognitive confidence; need to control thoughts; and cognitive self-consciousness. High scores reflect more reported problems with the item in question. Previous studies have found the psychometric properties to be good (Wells and Cartwright-Hatton, 2004).

CAS-1 (Wells, 2009) has four rating scales assessing general components of the cognitive attentional syndrome (CAS) and general positive and negative metacognitive beliefs. The instrument is typically used as a session to session instrument in MCT when no disorder-specific measure is appropriate. The first scale assesses time spent worrying and ruminating during the last week on a scale from 0 (no time) to 8 (all the time). The second scale measures threat monitoring in the same fashion. The third scale measures six examples of unhelpful coping behaviours, such as “avoid situations,” while the fourth scale assesses four examples of negative metacognitive beliefs (“I cannot control my thinking”) and four examples of positive metacognitive beliefs (“Worrying helps me cope”).

The Social Phobia Rating Scale (SPRS; Wells, 1997) has five rating scales assessing key components of one of the most commonly employed CT treatments for social phobia (Clark and Wells, 1995); distress, avoidance, self-consciousness, use of safety behaviours, and negative beliefs. In the present study, we used two of the subscales from the SPRS: (1) Use of safety behaviours; patients are asked to rate how often they use different types of safety behaviours (e.g., “try to relax”) when they have social anxiety on a scale ranging from 0 (not at all) to 8 (all the time). The subscale includes 15 items and therefore the total score range between 0 and 120. (2) Negative beliefs; the scale consists of 14 items (e.g., “I look bad”; “They will notice I’m anxious”), each item ranging from 0 to 100. This scale was used as measure of social phobic beliefs typical for social phobic patients, ranging from 0 to 1400. The psychometric properties of the SPRS have been reported as good (Nordahl et al., unpublished).

### Procedure

#### Assessment

Patients referred to the outpatient university clinic for treatment of social anxiety by their GP and other psychiatry services (e.g., the student’s mental health service) were invited to attend an assessment interview for possible participation in the current study. All patients were assessed by assessors who were trained in administering the Structured Clinical Interview for DSM-IV (SCID I and II). Patients completed the first battery of self-report measures before attending the assessment interview. The baseline period for included patients was a minimum of 3 weeks showing stable FNE-score (the primary outcome measure). Therefore, after the assessment interview the included patients rated themselves on the FNE and CAS-1 over the succeeding weeks. All patients had a stable FNE score over the three first consecutive weeks, and were therefore scheduled for treatment within a week after the third baseline measuring point. During treatment, the FNE and CAS-1 were completed before each session. A complete set of questionnaires was administered post-treatment and at 6 months’ follow-up. At post-treatment the SCID I and II was administered again by the same assessor who met the patient at pre-treatment.

#### Treatment

The treatment consisted of eight weekly sessions of 45–60 min duration and followed the generic MCT structure outlined by Wells (2009) and consisted of the following elements:

(1) A case formulation based on the generic metacognitive model was developed. This conceptualization emphasised the CAS as the primary maintenance factor of social anxiety, and showed how different metacognitive belief domains give rise to the CAS and how they block adaptive coping with social anxiety. Following the development of the case formulation, patients were socialised to the formulation in order to get a better understanding of how their social anxiety persists, and hence what should be the goals for treatment (abandon CAS strategies, explore and challenge metacognitive beliefs).
(2) The attention training technique (ATT) was introduced to facilitate a metacognitive mode of processing and to allow the patient to make discoveries about flexible executive control. The patients were asked to implement ATT twice a day for at least 4 weeks for homework, and in-session practise of the ATT was given in the first two sessions. The patients’ experiences with the technique were discussed, aiming to facilitate reduction of self-processing strategies and challenging metacognitive beliefs about the uncontrollability and danger of thoughts.
(3) Verbal reattribution strategies were used to modify negative beliefs concerning the uncontrollability of worry and rumination, and worry/rumination postponement was introduced to reduce the CAS and as an experiment to test false metacognitive beliefs about these processes being uncontrollable. Detached mindfulness was introduced with a link to ATT and presented as an alternative way to react to negative thoughts such as “What If I sound foolish” in preference to activation of the CAS.
(4) Threat monitoring was addressed, e.g., by an advantages-disadvantages analysis to address the process of constructing the observer perspective in social situations. The consequences of this strategy were highlighted, and positive metacognitive beliefs about constructing an inner image (e.g., “constructing an inner image of how I look helps me avoid making a bad impression on others”) were challenged.
(5) Two behavioural experiments in combination with Situational Attentional Refocusing (SAR) were conducted intended to counteract threat monitoring in social situations, and to facilitate adaptive information processing in a social setting. For example, the patient and therapist went for a 10-min walk. In the first half, the patients were told to be as self-conscious as possible, in the second half they were asked to switch their attention flexibly around and notice the surroundings. This experiment was used to enhance awareness over flexible attentional control, to highlight the consequences of self-consciousness and to challenge the patient’s positive beliefs about self-focused processing. Patients were asked to try and remember what they had noticed when self-conscious and when externally focused, the contrast in performance was used to challenge the patient’s belief that they had poor cognition. By attributing this to the attentional strategy they were choosing to engage in beliefs underlying low cognitive confidence could be challenged.
(6) Each patient was encouraged to apply their new awareness over flexible attentional control when facing challenging situations, and maladaptive coping strategies such as avoidance were briefly addressed with reference to its ability to prohibit the execution and discovery of adaptive metacognitive control. Worry and rumination were banned.
(7) Relapse prevention was implemented by making a therapy blueprint in the form of an “old plan –new plan.” Patients were encouraged to implement the new plan in future social situations to maintain and strengthen the gains made over the course of treatment.

#### Training

All patients were treated by the first author who is a clinical psychologist who has completed the MCT- Institute 2-year diploma and treatment was directed and supervised by Adrian Wells, the originator of MCT. Treatment used the techniques and structure as set out in a treatment manual (Wells, 2009).

### Data Analysis

The aim of single case research is to determine if there is a clear treatment effect following the introduction of the intervention, and hence if MCT could be a suitable treatment for SAD. Accordingly, visual examination of graphed data provides a stringent test of the treatment effects as only unambiguous effects will be apparent (Parsonson and Baer, 1992). Therefore, session by session scores across baseline, treatment and follow-up on the FNE and CAS (worry/rumination and threat monitoring) are illustrated. Descriptive statistics are presented for individual patients at pre-treatment, post-treatment and follow-up on the following measures: FNE, SAD, SIAS, BAI, BDI, MCQ-30, SPRS; social phobic beliefs, and SPRS; use of safety behaviours.

## Results

Pre-treatment, post-treatment and follow-up scores for each patient on standardised measures of social anxiety (FNE, SAD, SIAS) non-specific anxiety symptoms (BAI), depressive symptoms (BDI), metacognitive beliefs, use of safety behaviours and rating of social phobic beliefs are presented in **Table 1**. Eight sessions of MCT were associated with substantial reductions on all measures of social anxiety. At post-treatment, all patients were asymptomatic on BAI and BDI. Metacognitive beliefs were addressed in most treatment sessions, and decreased substantially from pre to post intervention. Finally, cognitive self-beliefs and use of safety behaviours showed a substantial decrease from pre to post-treatment, even though these components were not addressed in treatment. At 6 months’ follow up, treatment gains were largely maintained.

**Table 1:** Descriptive statistics for all the three cases at pre-treatment, post-treatment, and 6 months’ follow-up.

Measure	Patient 1			Patient 2			Patient 3		
	Pre	Post	FU	Pre	Post	FU	Pre	Post	FU
FNE	6	0	0	25	9	8	28	14	14
SAD	2	0	0	19	8	9	21	3	8
SIAS	15	3	6	49	21	17	61	23	28
BAI	8	0	3	16	2	3	11	0	9
BDI	6	0	2	24	2	4	28	4	10
MCQ-30	52	34	34	74	37	39	64	35	40
Social phobic beliefs	290	0	0	680	230	160	960	110	90
Use of safety behaviours	48	0	3	50	30	21	47	3	18

*FNE, Fear of Negative Evaluation; SAD, Social Anxiety and Distress scale; SIAS, Social Interaction and Anxiety Scale; BAI, Beck Anxiety Inventory; BDI, Beck Depression Inventory; MCQ-30, Metacognitions questionnaire 30*.

**FIGURE 1:**
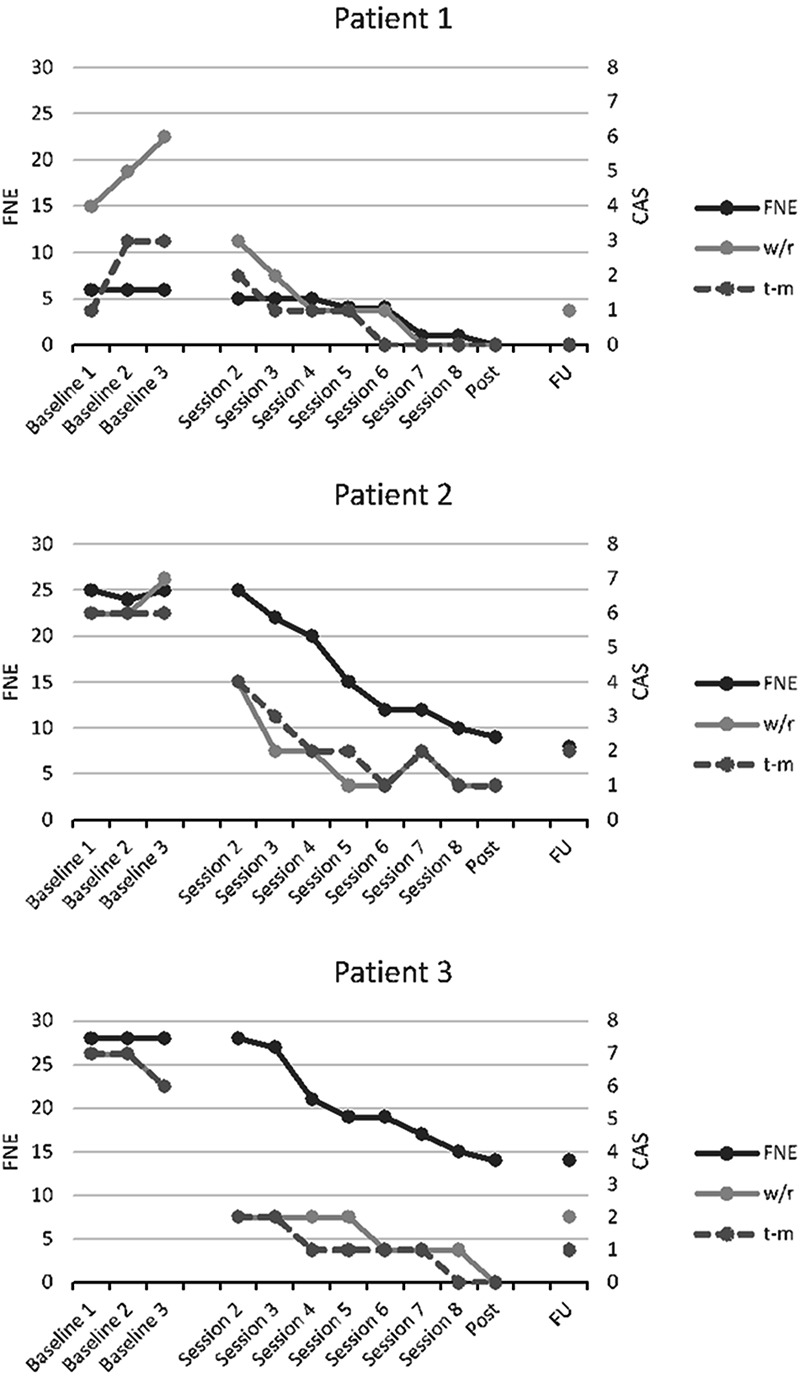
Scores on the Fear of Negative Evaluation (left *y*-axis), and time spent worrying/ruminating (w/r: CAS-1 item 1) and threat monitoring (t-m: CAS-1 item 2) the last week (right *y*-axis) across baseline, MCT and follow-up for each patient.

Each patient’s score on the Fear of Negative Evaluation (the primary outcome measure) and time spent worrying/ruminating and threat monitoring during the last week during the baseline, treatment and follow-up phase are illustrated in **Figure 1**. As can be seen, all patients showed a stable FNE score across the baseline period. Patient 1 presented with the performance subtype of SAD, which most likely is the reason why her FNE pre-treatment score was only six points. With the introduction of treatment, rapid and substantial reductions in CAS-activity can be observed in all three cases. The largest decrease in CAS activity was observed following the first two treatment sessions for all patients. The graphs also show that the FNE scores changed less rapidly than the CAS, but that they seem to follow the same trajectory. This result is consistent with the hypothesised effect of MCT on underlying process-related variables that are purported to subsequently impact on symptoms. Gains made during treatment were maintained through to the 6 months follow up point, with all patients having substantially lower FNE score at 6 months compared to baseline.

### Post-treatment Diagnostic Assessment

In addition to self-report measures, all patients were re-assessed with the SCID post-treatment by the same assessor they met before inclusion in the study. None of the patients met the diagnostic criteria for SAD following treatment. Patient 3, who prior to treatment also was diagnosed with comorbid major depressive disorder and AvPD, did not meet criteria for any diagnosis post-treatment.

## Discussion

The primary aim of the current study was to show any effects associated with generic MCT across the social anxiety continuum. Substantial reductions were obtained on all measures of social anxiety symptoms at post-treatment and at 6 months’ follow-up. Moreover, MCT seemed to be associated with change in underlying cognitive style (e.g., worry and self-focus attention) and metacognitive beliefs that according to the metacognitive model are implicated in the cause and maintenance of SAD (Wells and Matthews, 1994; Nordahl and Wells, 2017b).

Overall the treatment was well tolerated and none of the patients reported a worsening of symptoms or distress during the course of treatment. After eight treatment sessions, none of the patients fulfilled the criteria for a mental disorder, and treatment was associated with reductions in social anxiety, general anxiety symptoms, depressive symptoms, and metacognitive beliefs. Patient 1 who presented with the performance type of SAD, was asymptomatic after session 5, and could have terminated treatment at that point. Even though patient 1 presented with low scores on self-report symptom measures, she scored relatively high on self-report measures of maladaptive metacognitive beliefs. Low symptom scores can present a challenge to treat as they can be difficult to formulate outside of specific exposure to feared situations. However, the elevated metacognition scores may well be a marker for an underlying problem which remains latent until activated. Patient 3 showed a remarkable change during the treatment period, and no longer met the diagnostic criteria for major depressive disorder or AvPD post-treatment. While recovery from a personality disorder in only eight sessions is striking, similar tendencies have been reported by Hjemdal et al. (2017) in patients with major depressive disorder and comorbid disorders undergoing MCT for depression.

Interestingly, the present study showed that MCT was associated with substantial improvements for three patients with different presentations of SAD without addressing elements such as schemas, negative automatic thoughts or safety behaviours which are important factors in CBT. According to CBT models (Clark and Wells, 1995; Rapee and Heimberg, 1997), schemas and safety behaviours are central maintenance factors of self-focused attention and social anxiety symptoms. In the present study we found that cognitive belief ratings and use of safety behaviours decreased substantially, even though these factors were not addressed in treatment. This finding is interesting in light of the metacognitive model of psychological disorders (Wells and Matthews, 1994; Wells, 2009) which suggest that cognitive beliefs/schemas could be input and/or output of the cognitive attentional syndrome, but not a cause of psychological disorder. In a recent study by Nordahl et al. (2017), change in social phobic beliefs was not a significant a predictor of symptom improvement following treatment for SAD, while change in self-consciousness and change in negative metacognitive beliefs were. Likewise, safety behaviours may be a consequence of the CAS (e.g., worrying), and not the direct cause of social anxiety.

The results from this study are encouraging; however, this study is only based on three cases with different presentations of SAD which limits inferences about the generalizability of treatment effects. Whilst the multiple baseline design controls for effects such as time, we are unable to partial-out the effects specifically due to metacognitive treatment techniques as opposed to non-specific factors. Moreover, although outcome was measured each week, more frequent measurements and the use of experience sampling methods could reveal greater dynamics in the data. The use of only one therapist means that it is not possible to determine the influence of factors such as skill level. Another limitation is that the assessors were not blind to the presence/absence of treatment which may have influenced the assessor ratings. Moreover, the treatment delivered in the current study was based on generic MCT-principles informed by recent research within the field of MCT and SAD, rather than a disorder specific MCT-manual which potentially could make treatment more efficacious.

## Conclusion

Metacognitive Therapy was associated with substantial improvement in social anxiety and seemed to be associated with changes in underlying cognitive style (the CAS) and metacognitive beliefs. The results are preliminary and based on a case series with no control over non-specific factors or spontaneous recovery, but the results are indicative of the potential usefulness of MCT for SAD and support further evaluation in this context.

## Author Contributions

HN treated all the patients and wrote the first draft of the manuscript. AW provided supervision throughout the treatment and the research process, and contributed substantially to the final version of the manuscript.

## Conflict of Interest Statement

The authors declare that the research was conducted in the absence of any commercial or financial relationships that could be construed as a potential conflict of interest.
